# Association of measures of central fat accumulation indices with body fat distribution and metabolic, hormonal, and inflammatory parameters in women with polycystic ovary syndrome

**DOI:** 10.20945/2359-3997000000157

**Published:** 2019-07-11

**Authors:** Victor Barbosa Ribeiro, Gislaine Satyko Kogure, Iris Palma Lopes, Rafael Costa Silva, Daiana Cristina Chielli Pedroso, Rui Alberto Ferriani, Cristiana Libardi Miranda Furtado, Rosana Maria dos Reis

**Affiliations:** 1 Universidade de São Paulo Departamento de Ginecologia e Obstetrícia Faculdade de Medicina de Ribeirão Preto Universidade de São Paulo Ribeirão Preto SP Brasil Departamento de Ginecologia e Obstetrícia, Faculdade de Medicina de Ribeirão Preto, Universidade de São Paulo (FMRP-USP), Ribeirão Preto, SP, Brasil; 2 Instituto Federal de São Paulo Instituto Federal de São Paulo Jacareí SP Brasil Instituto Federal de São Paulo, Jacareí, SP, Brasil

**Keywords:** Polycystic ovary syndrome, visceral fat, adiposity, lipid accumulation product, body composition, dual X-ray absorptiometry, body fat distribution

## Abstract

**Objective:**

To investigate the associations among visceral adiposity index (VAI), lipid accumulation product (LAP), body fat percentage (%), and android/gynoid ratio (A/G ratio) in women with polycystic ovary syndrome (PCOS) and verify if the parameters representative of visceral obesity correlate with and exhibit the same frequency as body composition variables; anthropometric indices; and metabolic, hormonal, and inflammatory parameters.

**Subjects and methods:**

This was a cross-sectional study that included 94 women with PCOS. Hormonal, metabolic, and inflammatory parameters were analyzed in all women. Free androgen index (FAI) and homeostasis model assessment (HOMA-IR), as well as LAP, VAI, and anthropometric indices, were calculated. The regions of interest (ROIs) in body composition and body composition indices were evaluated using a dual X-ray absorptiometry (DXA). Overall, 32 variables were selected as markers of body fat distribution.

**Results:**

Among the 32 markers evaluated, 29 correlated with LAP, whereas 25 correlated with VAI, 19 with body fat (%), and 30 with A/G ratio. Additionally, some markers correlated with the four adiposity indices evaluated: ROIs, except for total mass and leg fat (%); body composition (body mass index, waist circumference, and hip circumference) indices; fasting insulin; and C-reactive protein.

**Conclusion:**

LAP and VAI may be sensitive measures for screening and preventing metabolic syndrome and insulin resistance in PCOS, with LAP being more sensitive than VAI, and the A/G ratio may be more sensitive than body fat percentage.

## INTRODUCTION

Polycystic ovary syndrome (PCOS) is an endocrine disorder affecting approximately 5-16% of women in reproductive age and is characterized by at least two of the following conditions: hyperandrogenism (clinical or biochemical), irregular menstrual cycle, and presence of ovarian cysts (1,2). PCOS directly affects body composition and anthropometric indices, as well as endocrine, metabolic, and cardiovascular system parameters (1-5). Central obesity is also a prevalent characteristic because of hyperandrogenism and insulin resistance (IR), which act in vicious feedback mechanisms (6,7). However, abdominal fat may go undetected in women of normal weight who exhibit an apparently lean PCOS (6,7), predisposing them to risk factors for chronic diseases, such as metabolic syndrome and arterial hypertension (1,4-5). The prevalence of metabolic syndrome among individuals with PCOS is 12.6%, which is almost seven times higher than that in healthy women with a higher body mass index (BMI) (8). The prevalence of IR (14.2% vs. 9.3%) (8) and non-alcoholic fatty hepatitis are also higher (23.8% vs. 3.3%) (9).

Different methods are utilized to evaluate body fat distribution for the screening of cardiometabolic risk. Dual X-ray absorptiometry (DXA) has been considered as a reference standard (10), whereas anthropometric methods and biochemical examinations in the lipidogram area are considered the cheapest and simplest standards (1,3,11). Recently, mathematically determined indices, such as visceral adiposity index (VAI) and lipid accumulation product (LAP), have been deemed effective (12-14). These methods are considered more economical and practical options, as they present formulas, anthropometric indices, and biochemical measures that allow clinical practice and research to investigate better tracking and prevention (13-15).

This study aimed to investigate the associations among VAI, LAP, total body fat percentage, and the A/G ratio and to verify if visceral obesity parameters correlate and with the same frequency as variables of body composition, anthropometric indices, and metabolic, hormonal, and inflammatory parameters.

## SUBJECTS AND METHODS

This study was approved by the Institutional Review Board of the University Hospital, Ribeirao Preto Medical School, University of Sao Paulo (Protocol number nº 9640/2014), and all participants provided written informed consent. The authors confirm that all ongoing and related trials for this intervention were registered in the Brazilian Clinical Trials Registry (ReBec; RBR-78qtwy) and International Controlled Randomized Controlled Trial (ISRCT) Registry 10416750.

### Subjects

A total of 110 consecutive women, aged 18-39 years, with PCOS and BMI of 18-39.9 kg/m^2^ and who were not engaged in regular and systematic physical exercise, were recruited. Women were selected regardless of race, parity, or social class. PCOS diagnosis was made according to Consenso de Rotterdam (1). Participants were selected at the Outpatient Clinic of the Human Reproduction Sector, Department of Gynecology and Obstetrics at the Faculty of Medicine of Ribeirão Preto (DGO/HCFMRP; University of São Paulo, Ribeirão Preto, São Paulo, Brazil). Exclusion criteria included the presence of systemic diseases, use of medications that interfere with the hypothalamic-pituitary-ovarian axis, pregnancy, smoking, musculoskeletal disorders, hypothyroidism, hyperprolactinemia, Cushing’s syndrome, and congenital adrenal hyperplasia. Participants received transvaginal pelvic ultrasound examinations using the Voluson E8 Expert machine (GE HealthCare, Zipf, Austria) to evaluate the presence of polycystic ovaries. To diagnose PCOS, peripheral blood samples were collected and thyroid-stimulating hormone (TSH), 17-hydroxyprogesterone (17-OHP), prolactin, and testosterone concentrations were measured.

### Blood dosages

In the present study, 20.0 mL of whole blood was collected until the eighth day of the menstrual cycle (early follicular phase) or any day when the participant experienced amenorrhea. Endocrine assessments were performed by measuring serum testosterone and androstenedione levels using chemiluminescence (Immulite 1000, Siemens). Luteinizing hormone (LH), follicle-stimulating hormone (FSH), estradiol (E2), TSH, 17-OHP, prolactin (PRL), sex hormone-binding globulin (SHBG), homocysteine, and C-reactive protein levels and glucose metabolism were assessed by measuring fasting insulin levels using chemiluminescence (Immulite 2000, Siemens). Fasting glycemia was determined using the oxidase method. Total cholesterol (TC), high-density lipoprotein (HDL) cholesterol, low-density lipoprotein (LDL) cholesterol, and triglyceride (TG) levels were assessed using the enzymatic method. FAI was calculated using the following formula: total testosterone (nmol/L)/SHBG (nmol/L) × 100. Total testosterone was calculated by multiplying the value obtained (in ng/dL) with the conversion factor 0.0347 (16). To detect IR, the index given by the homeostatic model assessment (HOMA-IR) was calculated using the following equation: [(fasting glycemia in mg/dL × 0.05551) × fasting insulin in μU/mL]/22.5 (17).

### Visceral adiposity index and lipid accumulation product

VAI was measured using the formula [waist circumference (WC, cm)/36.58 + (1.89 × BMI) × (TG/0.81) × (1.52/HDL) (13). Similarly, LAP was calculated using the formula [{WC (cm) - 58} × TG (nmol/L)] (14).

### DXA measurements

Body composition was assessed using DXA scanning (Hologic 4500 device QDR Discovery^®^ Series – Waltham: MA, USA), with a full-body scan. Analysis was performed using the 5 Discovery Wi model software (version 13.0). The two ROIs from the scan used in this analysis were the total mass (mass of fat (g) plus lean mass, including bone mineral content (g) and fat percentage (fat mass/total mass × 100), which included the mass of the head, arms, legs, trunk, body, and the android and gynoid regions. Arm and leg fat was defined as the sum of fat percentage in two arms and both legs, divided by two. The following indices were calculated according to fat distribution: A/G ratio, fat mass/height^2^ (kg/m^2^), and percentage of fat in the trunk/percentage of fat in the legs (% fat trunk/% fat legs) (18).

### Anthropometry

Body weight and height (alt) were evaluated using a Filizola Brasil platform scale with 0.1-kg and 0.5-cm precision. WC was measured with the individual standing with arms at the side of the body, feet together, and relaxed abdomen; a horizontal measurement was obtained on the narrower part of the dorsum (above the navel and below the xiphoid process). The hip circumference (HC) was measured from the same position in the region with the largest circumference of the buttock (15). The waist-hip ratio (WHR) was calculated by dividing WC (cm) by HC (cm) (15). BMI (in kg/m^2^) was calculated by dividing the body weight by the square of height. Measurements were made using a tape anthropometric metal of Sanny brand, and IMC was defined as weight (kg)/height^2^.

### Statistical analyses

Statistical analyses were performed using Sigma Stat software (version 11.0) (Systat Software Inc., San Jose, CA, USA). The Shapiro-Wilk test was performed to evaluate the normal distribution of variables. The between-group comparisons, with different BMI measurements (normal (n = 28), overweight (n = 28), and obesity (n = 38), were investigated using a one-way analysis of variance test followed by the post hoc Student-Newman-Keuls multiple comparison test. The results were expressed as mean ± standard deviation. Correlation analysis was performed using the Pearson correlation and linear regression for parametric variables and Spearman’s rank-order correlation for non-parametric variables. R-values were obtained and correlations were analyzed using the following specifications: 0-0.19, very low correlation; 0.20.39, low; 0.4-0.59, moderate; 0.6-0.79, high; 0.8-0.99, very high; and 1.0, perfect correlation (19). *P*-values of < 0.05 were considered statistically significant.

## RESULTS

Sixteen participants were excluded because they had not been diagnosed with PCOS. Therefore, 94 participants were investigated and classified according to BMI as follows: 28 with normal weight (18.0-24.9 kg/m^2^), 28 overweight (25.0-29.9 kg/m^2^), and 38 obese (>30.0 kg/m^2^). The age (normal BMI, 27.0 ± 5.18 years; overweight, 28.0 ± 5.10 years; obese 29.9 ± 5.18 years) and height (normal BMI, 1.62 ± 0.06 meters; overweight, 1.62 ± 0.06 meters; obese, 1.62 ± 0.08 meters) were similar between groups.

The endocrine, metabolic, and inflammatory characteristics are summarized in Table 1. SHBG levels were significantly higher among the normal group, in relation to the overweight group (p = 0.028) and lower triglycerides (p = 0.044), fasting insulin levels (p = 0.005), and HOMA and FAI scores (p = 0.005, both) were observed when comparing these groups. When comparing the normal and obese groups, differences in the mean SHBG and HDL levels were significant, which were lower in the obese group (p < 0.001 and p = 0.007, respectively). Additionally, fasting insulin levels and HOMA-IR scores were higher in the obese group (p < 0.001, both), and this group also presented lower 17-OHP values than the overweight (0.016) and normal (0.001) groups.

**Table 1:** Hormonal, metabolic and inflammatory parameters according to the body mass index (normal, overweight, obesity)

	Normal (n = 28)	Overweight (n = 28)	Obesity (n = 28)
	
	Media (SD)	Media (SD)	Media (SD)
**Sexual Hormones**			
PRL, ng/mL	16.6 (9.24)	14.32 (7.72)	12.07 (6.82)
17-OHP, ng/dL	119 54)	103 (65)	71 (40)**^#^
Testosterone, ng/dL	111 (52)	100 (52)	105 (49)
Androstenedione, ng/dL	81 (57)	78 (39)	76 (53)
SHBG, nmol/L	61 (33)	43 (25)*	39 (24)^#^
FAI	7.09 (4.01)	10(10.1)*	12.4 (8.9)^#^
E2, pg/mL	69 (60)	60 (44)	48 (19)
LH, uUI/mL	9.87 (8.79)	8.28 (4.70)	7.48 (4.22)
FSH, uIU/mL	5.76 (3.31)	5.08 (2.00)	5.36 (1.65)
**Metabolic Parameters**			
TSH, uIU/mL	2.00 (1.05)	2.36 (1.18)	2.41 (1.30)
Total Cholesterol, mg/dL	175 (36)	181 (29)	189 (33)
Triglycerides, mg/dL	79 (32)	148 (169)*	134 (78)
HDL, mg/dL	52 (12)	48 (11)*	44 (9,0)^#^
LDL, mg/dL	109 (33)	107 (23)	118 (24)
Fasting Glycemia, mg/dL	81 (6)	85 (12)	84 (11)
Fasting Insulin, mg/dL	6.37 (6.23)	11.0 (6.77)*	13.5 (7.25)^#^
HOMA-IR	1.25 (1.14)	2.40 (1.70)*	2.91 (1.88)^#^
**Inflammatory Parameters**			
Homocysteine, µmol/L	7.35 (1.98)	6.52 (2.12)	7.45 (2.08)
C-Reactive Protein, mg/dL	0.22 (0.29)	0.30 (0.25)	0.65 (0.45)

SD: Standard deviation; 17-OHP: 17-hydroxyprogesterone; PRL: prolactin; SHBG: sex hormone binding globulin; FAI: free testosterone index; estradiol: E2; TSH: thyroid stimulating hormone; 17-OHP: 17-hydroxyprogesterone; PRL: prolactin; LH: luteinizing hormone; FSH: follicle stimulating hormone; HDL: high density lipoproteins; LDL: low density lipoproteins; HOMA-IR: homeostatic model assessment.

* p < 0.05 (normal BMI group vs overweight BMI group); ** p < 0.05 (group BMI overweight vs group BMI obesity); ^#^ p < 0.05 (group BMI normal vs group BMI obesity).

As shown in Table 2, LAP values were higher in the overweight and obese groups that those in the normal group (p = 0.003 and p < 0.001, respectively), and the obese group exhibited higher LAP values than the overweight group (p = 0.003). Moreover, the VAI was higher in both overweight and obese groups (p = 0.027 and p < 0.001, respectively). When analyzing the ROIs according to BMI (Table 2), the arm total mass, trunk total mass, leg total mass, body total mass (p < 0.001, all), arm total mass (p = 0.043), trunk fat (%), and body fat (%) (p = 0.01, both), as well as fat mass/height^2^ (p < 0.001) and A/G ratio (p = 0.046) were progressively increasing from the normal group to the overweight group. When comparing the normal and obese groups, significant differences were detected in the arm total mass, trunk total mass, trunk fat (%), leg total mass, body total mass, body fat (%) (p < 0.001, all), and arm total mass (p = 0.003), as well as in fat mass/height^2^, A/G ratio, and % fat trunk/% fat legs (p < 0.001, all).

**Table 2:** Anthropometric indices, body composition and body composition indices, according to body mass index (normal, overweight, obesity)

	Normal (n = 28)	Overweight (n = 28)	Obesity (n = 28)
	
	Media (SD)	Media (SD)	Media (SD)
**Anthropometric Index**			
BMI	22.6 (1.49)	27.2 (1.41)*	34.2 (2.62)**^#^
WC, cm	75 (5.2)	86 (6.0)*	100 (7.4)**^#^
HC, cm	93 (18.8)	104 (5.7)*	115 (6.8)**^#^
WHR	0.78 (0.05)	0.83 (0.06)*	0.87 (0.07)**^#^
**Adiposity Index**			
VAI	2.73 (1.55)	5.45 (6.27)*	6.26 (4.45)^#^
LAP	14.4 (11.7)	39.8 (35.4)*	52.9 (36.8)**^#^
**Body Composition**			
TM arm (g)	5292 (1238)	7376 (1118)*	8911 (2196)**^#^
MF arms (%)	42.6 (5.5)	44.9 (5.4)	48.6 (6.2)**^#^
TM trunk (g)	26473 (4089)	33560 (4074)*	43123 (5409)**^#^
TF trunk (%)	3.,9 (5.7)	40.0 (4.4)*	43.6 (4.6)**^#^
TM leg (g)	21362 (2852)	24623 (3287)*	31202 (5334)**^#^
MF leg (%)	42.2 (4.5)	43.3 (5.9)	44.9 (6.0)
TM body (g)	58630 (6186)	69203 (10475)*	89170 (12286)**^#^
TF body (%)	37.2 (3.84)	40.6 (4.26)*	43.6 (4.43)**^#^
**Body Composition Index**			
TF trunk/leg	0.83 (0.14)	0.93 (0.11)*	0.98 (0.11)^#^
FM/height^2^ (kg/m^2^)	8.29 (1.18)	10.9(1.52)*	14.7 (1.99)**^#^
A/G ratio	0.87 (0.13)	0.97 (0.11)*	1.03 (0.09)**^#^

SD: standard deviation; %: percentage; A/G ratio: android/gynoid fat ratio; VAI: visceral adiposity index; LAP: lipid accumulation product; BMI: body mass index; cm: centimeters; WC: waist circumference; HC: hip circumference; WHR: hip waist ratio; TM: total mass; MF: mean fat; TF: total fat; g: gram; FM: fat mass; (kg/m^2^), Kilogram/square meter. * p < 0.05 (normal BMI group vs overweight BMI group); ** p < 0.05 (group BMI overweight vs group BMI obesity); ^#^ p < 0.05 (group BMI normal vs group BMI obesity).

Among the 32 markers evaluated, LAP values correlated with 25, whereas VAI correlated with 23 markers. Conversely, total body fat correlated with 16 markers, whereas the A/G ratio correlated with 26.

### Correlation between LAP, VAI, A/G ratio, and total body fat percentage

**Figure 1:**
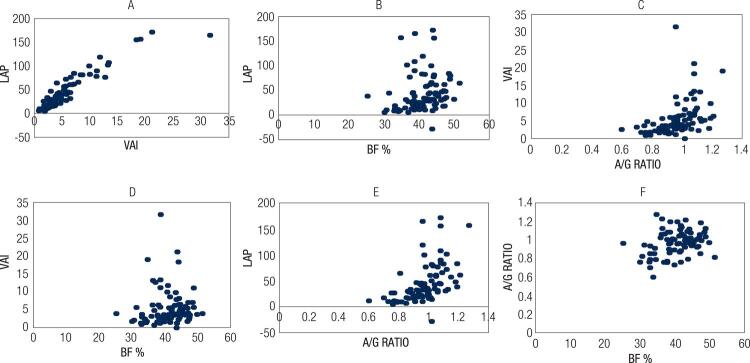
Correlation between visceral adiposity index (VAI), lipid accumulation product (LAP), total fat percentage, android/gynoid ratio.

The correlations among LAP, VAI, percentage of total fat, and A/ G ratio are shown in Figure 1A. LAP was very highly correlated (p < 0.05) with VAI, moderately with A/G ratio, and weakly with body fat. In addition, VAI was positively, moderately correlated (p < 0.05) with the A/G ratio and a very weakly correlated with body fat; the A/G ratio was weakly correlated with body fat (Figure 1).

### Correlation between adiposity indices, biochemical parameters, anthropometric indices, and body composition

In this study, VAI was weakly, negatively correlated with SHBG levels, but moderately, positively correlated with FAI scores in endocrine parameters. Furthermore, VAI was weakly, positively correlated with TC, LDL, fasting glycemia, fasting insulin levels, and HOMA-IR score. Conversely, VAI was very highly, positively correlated with TG levels, but moderately, negatively correlated with HDL level for the metabolic parameters, and weakly, positively correlated with the C-reactive protein level for the inflammatory parameters. (Table 3). Regarding the anthropometric indices, VAI was weakly, positively correlated with BMI and HC, and moderately, positively correlated with WC and WHR. Among the body composition and body composition indices, VAI showed a low correlation with total body mass, trunk total mass, body mass, arm fat (%), and trunk fat (%), as well as a moderate and positive correlation with fat mass/height^2^. VAI was weakly, positively correlated the % fat trunk/% leg fat ratio (Table 4).

**Table 3:** Correlation of total fat percentage, android/gynoid fat ratio, visceral adiposity index (VAI) and lipid accumulation product (LAP) with sexual hormones, metabolic parameters and inflammatory parameters of women with Polycystic Ovarian Syndrome (N = 94)

	Mean (SD)	Fat body (%)		A/G Ratio		VAI		LAP	

		R	*p*	R	*p*	R	*p*	R	*p*
**Sexual Hormones**									
Testosterone, ng/dL	105 (51)	- 0.142	0.172	- 0.028	0.782	0.017	0.868	- 0.001	0.974
Androstenedione, ng/dL	78 (50)	-0.040	0.704	- 0.185	0.075	-0.135	0.193	- 0.160	0.123
SHBG, nmol/L	47 (28)	-0.175	0.091	- 0.432*	< 0.001	-0.313*	< 0.001	- 0.484*	< 0.001
FAI	10.4 (8.4)	0.052	0.616	0.226*	< 0.001	0.228*	0.030	0.324*	< 0.001
E2, pg/mL	58 (43)	- 0.071	0.495	- 0.212*	0.040	-0.077	0.460	- 0.166	0.111
LH, uUI/mL	8.43 (6.08)	- 0.039	0.706	- 0.019	0.852	-0.106	0.310	- 0.082	0.432
FSH, uIU/mL	5.40 (2.34)	- 0.048	0.647	0.112	0.281	0.015	0.888	0.073	0.483
**Metabolic Parameters**									
Total Cholesterol, mg/dL	182 (33)	0.188	0.070	0.361*	< 0.001	0.306*	< 0.001	0.385*	< 0.001
Triglycerides, mg/dL	122 (109)	0.150	0.148	0.525*	< 0.001	0.940*	< 0.001	0.844*	< 0.001
HDL, mg/dL	48 (11)	0.001	0.929	-0.273*	< 0.001	- 0.533*	< 0.001	- 0.353*	< 0.001
LDL, mg/dL	112 (27)	0.189	0.068	0.355*	< 0.001	0.229*	0.026	0.245*	0.020
Fasting Glycemia, mg/dL	83 (10)	- 0.041	0.691	0.265*	< 0.001	0.332*	< 0.001	0.353*	< 0.001
Fasting Insulin,mg/dL	10.66 (7.38)	0.249*	0.016	0.532*	< 0.001	0.354*	< 0.001	0.538*	< 0.001
HOMA-IR	2.27 (1.76)	0.219*	0.034	0.540*	< 0.001	0.390*	< 0.001	0.558*	< 0.001
**Inflammatory Parameters**									
Homocisteyne, µmol/L	7.14 (2.08)	0.004	0.972	-0.173	0.096	-0.032	0.756	-0.026	0.0801
C-Reactive Protein, mg/dL	0.42 (0.40)	0.420*	< 0.001	0.516*	< 0.001	0.356*	< 0.001	0.513*	< 0.001

SD: deviation (SD); R: correlation index; SHBG: sex hormone binding globulin; FAI: free testosterone index; E2: estradiol; LH: luteinizing hormone; FSH: follicle stimulating hormone; HDL: high density lipoproteins; LDL: low density lipoproteins; HOMA-IR: homeostatic model assessment. * p < 0.05.

**Table 4:** Correlation of total fat percentage, android/gynoid fat ratio, visceral adiposity index (VAI) and lipid accumulation product (LAP) with anthropometric indices and body composition of women with polycystic ovarian syndrome (N = 94)

	Mean (SD)	Fat body (%)		A/G ratio		VAI		LAP	

		R	p	R	p	R	p	R	p
**Anthropometric Index**									
BMI	28.7 (5.3)	0.646*	< 0.001	0.558*	< 0.001	0.396*	< 0.001	0.711*	< 0.001
WC, cm	88 (15)	0.561*	< 0.001	0.600*	< 0.001	0.482*	< 0.001	0.789*	< 0.001
HC, cm	106 (15)	0.694*	< 0.001	0.317*	< 0.001	0.233*	0.024	0.561*	< 0.001
WHR	0.83 (0.07)	0.175*	0.092	0.637*	< 0.001	0.546*	<0.001	0.693*	< 0.001
**Body Composition**									
TM arm (g)	7376 (2235)	0.468*	< 0.001	0.402*	< 0.001	0.247*	0.016	0.539*	< 0.001
MF arms (%)	46 (6)	0.815*	< 0.001	0.261*	0.011	0.157	0.131	0.318*	< 0.001
TM trunk (g)	35315 (8407)	0.534*	< 0.001	0.534*	< 0.001	0.390*	< 0.001	0.707*	< 0.001
TF trunk (%)	40 (6)	0.563*	< 0.001	0.561*	< 0.001	0.231*	0.025	0.459*	< 0.001
TM leg (g)	26311 (5901)	0.573*	< 0.001	0.237*	0.021	0.190	0.066	0.502*	< 0.001
MF leg (%)	44 (6)	0.742*	< 0.001	- 0.187*	0.070	-0.168	0.105	-0.023	0.826
TM body (g)	74125 (16300)	0.563*	< 0.001	0.451*	< 0.001	0.333*	< 0.001	0.656*	< 0.001
**Body Composition Index**									
TF Trunk/Leg	0.92 (0.13)	0.253*	0.014	0.888*	< 0.001	0.510*	< 0.001	0.605*	< 0.001
FM/weight^2^ (kg/m^2^)	12.06 (4.99)	0.794*	< 0.001	0.530*	< 0.001	0.369*	< 0.001	0.662*	< 0.001

SD: standard deviation; R: correlation index; %: percentage; A/G ratio: android/gynoid fat ratio; VAI: visceral adiposity index; LAP: lipid accumulation product; BMI: body mass index; %: percentage; cm: centimeters; WC: waist circumference; HC: hip circumference; WHR: hip waist ratio; TM: total mass; MF: mean fat; TF: total fat; g: gram; FM: fat mass; (kg/m^2^), kilogram/square meter. * p < 0.05.

LAP was moderately (negatively) correlated with SHBG levels and moderately (positively) correlated with FAI scores for endocrine parameters; this correlation was low and positive with TC, LDL, and fasting plasma glucose levels, and moderate and positive with fasting insulin level and HOMA-IR score. Furthermore, these values were positively and very highly correlated with TG levels and negatively and weakly correlated with HDL levels for the metabolic parameters, whereas a moderate, positive correlation was maintained with the C-reactive protein level in regard to inflammatory parameters (Table 3). In the anthropometric indices, LAP was positively correlated with HC, BMI, WC, and WHR, but moderately with HC and highly with BMI, WC, and WHR. Regarding body composition and body composition indices, LAP was weakly correlated with arm fat (%), moderately and positively associated with trunk fat (%) and total arm mass trunk, and positively and highly correlated with both fat mass/height^2^ and % fat trunk/% leg fat (Table 4).

In relation to the body fat, no correlations were observed in the endocrine parameters; however, a low, positive correlation was observed between the fasting insulin level and metabolic parameters, and a moderate, positive correlation with C-reactive protein level for inflammatory parameters (Table 3). In the anthropometric indices, body fat was moderately, positively correlated with WC and highly, positively associated with BMI and HC. Regarding body composition and body composition indices, the body fat percentage was moderately correlated (P < 0.05) with the total arm mass and total body mass; positively, highly correlated with arm fat (%); positively, weakly associated with % fat trunk/% leg fat; and positively, highly correlated with fat mass/height^2^ (Table 4).

The A/G ratio was negatively, moderately correlated with SHBG level; moderately, positively correlated with FAI scores; and weakly, negatively associated with estradiol levels in the endocrine parameters. Furthermore, it was weakly, positively correlated with TC, LDL, and fasting glycemia levels, but moderately, positively correlated with TG, fasting insulin levels, and HOMA-IR score. The A/G ratio weakly, negatively associated with the HDL level in metabolic parameters, but moderately, positively correlated with the C-reactive protein level in the inflammatory parameters (Table 3). In the anthropometric indices, the A/G ratio was weakly, positively correlated with HC; moderately, positively correlated with BMI; and highly, positively associated with WC and WHR. In the body composition and body composition indices, the A/G ratio was weakly, positively correlated with arm fat (%) and leg total mass; positively, moderately related with trunk fat (%), total arm mass, trunk mass, and total body mass; very highly, positively correlated with fat mass/height^2^; and moderately, positively associated with % fat trunk/% leg fat (Table 4).

## DISCUSSION

The main finding of this cross-sectional study was that the parameters representative of visceral obesity were significantly associated with each other and phenotypic characteristics of PCOS. In general, a cascade effect was noted in women with PCOS where increased central fat influences the increase in insulin resistance. Conversely, insulin resistance interferes hypeandrogenism, with a feed and feedback circuit (20-22). The increase in these clinical factors may directly interfere the inflammation that may be more pronounced in PCOS (22).

There is no known literature identifying if the indices evaluated are associated with underlying markers of abdominal obesity, metabolic and endocrine disorders, and body fat distribution in women with PCOS. In recent years, VAI and LAP have been reported to express cardiometabolic risk (13-14) and are viable indices in risk identification, including PCOS (13,23). VAI and LAP indices were evaluated in individuals with type II diabetes and PCOS and were associated with IR; therefore, they were accredited in measuring IR risk in the absence of metabolic syndrome characteristics (11,14,24,25). Another study illustrated that LAP ≥ 34 and VAI ≥ 1.32 may be associated with metabolic syndrome, concomitant with PCOS (13). These indices are composed of markers such as TG, CC, and HDL that relate to each other and can present valuable information at low cost and accessibility (26).

In the present study, since the visceral fat may increase in non-obese women with PCOS (6-7), the participants were classified according to BMI. In accordance with the literature, increase in body composition variables and anthropometric indices was progressive with increased BMI (6,27). Fasting insulin levels and HOMA-IR score increased and SHBG levels decreased as BMI increased, possibly indicating impairment in the endocrine-metabolic parameters in PCOS as adiposity increases. This finding is similar to the results of the previous studies (28,29). LAP and VAI values increased as BMI increased. In standard operating procedures (SOPs), BMI-marked obesity is associated with increased risk of changes in LAP (over eight-fold) (28) and VAI levels are higher in overweight and obese subjects (29).

In the present study, both VAI and LAP values were found to be positively associated with A/G ratio and that only LAP values were positively associated with body fat percentage. However, surrogate indices, both VAI and LAP, can accurately differentiate visceral adiposity and subcutaneous adiposity (30), particularly VAI values (31). In accordance with previous findings by Mario and cols., although both LAP and VAI were positively correlated with HOMA-IR, the LAP correlation was higher than that of VAI. Another previous study indicated that both LAP and VAI are the best indices to predict IR in PCOS, as they both have a high sensitivity index (23). In addition, the A/G ratio was moderately, positively correlated with HOMA-IR scores. In 2016, Bouchi and cols. (32) suggested that the A/G ratio reflects peripheral insulin resistance because the A/G ratio was correlated with visceral fat accumulation measured using computed tomography (CT). Another important finding was the association of parameters representing visceral obesity with C-reactive protein levels, since obesity and IR contribute to the low-grade chronic inflammatory state in SOP (33). Although the correlation coefficients observed in this study were considered weak, the results align with other findings in women with PCOS (29) and individuals with type II diabetes (34).

Previous studies have also investigated the relationship between LAP and VAI with metabolic and hormonal parameters (23,35). Ramezani and cols. (36) reported that VAI and LAP was very highly, positively correlated with TG levels and moderately, negatively correlated with HDL, aligning with results of this study. Nonetheless, the findings in this study illustrate a weak, negative correlation of HDL with LAP values and the A/G ratio, as well as a moderate, positive correlation with TG levels. Conversely, Polyzos and cols. (35) reported that LAP was weakly, positively correlated with testosterone and fasting glycemia levels, moderately associated with FAI, and highly associated with HOMA-IR scores and fasting insulin levels. Further, a moderate, negative correlation was seen with SHBG levels, and Androulakis and cols. (37) reported a weak, positive correlation of VAI with fasting glycemia, FAI, estradiol, LDL and TC. In their study, a negative association was observed with SHBG levels and a moderate association was seen with fasting insulin levels and HOMA-IR scores. In the present study, FAI was weakly, positively correlated with A/G ratio, VAI, and LAP and negatively correlated with SHBG levels. These correlations are indicative of underlying conditions that increase the risk in women with PCOS, because increased free testosterone levels contribute to increased fasting insulin levels and liver fat resistance (6-7).

The correlations observed between LAP, VAI, and A/G ratio, as well as with metabolic, anthropometric, inflammatory, and hormonal predictors that interfere with different comorbidities suggest that LAP and VAI are integral in assessing body fat distribution (12,13). Evaluating the correlations of variables related to fat distribution and body composition indices, in addition to the anthropometric indices, indicated correlations with the percentage of total fat, A/G ratio, VAI, and LAP, which occurred in 12, 12, 11, and 12 cases, among the 13 cases, respectively. In addition, the percentage of total fat and LAP presented a greater number of moderate, high, and very high correlations, occurring in 11 of them, whereas correlations of this type only occurred in nine and three cases using A/G ratio and VAI, respectively. However, when correlations of the variables relating to fat distribution, body composition, and anthropometric indices were evaluated, these correlations occurred in 11 of 13 and 12 of 13 markers with VAI and total fat percentage, respectively; and occurred with A/G ratio and LAP as well. In addition, the percentage of total fat and LAP presented 11 moderate, high, and very high type correlations, whereas correlations of this type only occurred in nine and three cases for A/G ratio and VAI, respectively. LAP had correlations with more variables related to body fat distribution and exhibited stronger correlations than those presented by VAI. This finding suggests that for this type of evaluation, LAP is more reliable than VAI. However, both are low-cost, noninvasive, accessible, and predictive methods of metabolic alteration in women with PCOS (13,23,35).

In general, the body fat percentage, A/G ratio, VAI, and LAP are important measurements that correlate with several risk markers for chronic diseases and are generally altered in women with PCOS (2). In addition, the present results suggest that, for correlations involving endocrine-metabolic and inflammatory parameters, body fat percentage should be the last alternative among the four indices evaluated. When correlations involve the body fat distribution, body fat percentage and LAP are suggested to be chosen first.

This study had some limitations. DXA, which was used to measure body composition and body fat distribution, and other imaging technologies, such as CT and magnetic resonance imaging (MRI), should be used to assess visceral adiposity, due to their accuracy (38). Although DXA cannot distinguish between intra-abdominal and subcutaneous fat, research shows strong correlations between trunk fat mass measured with DXA and intra-abdominal fat measured with CT or MRI (39). In addition, although results indicate strong associations and influences among variables, causation cannot be implied. An inference from this study is inherent to a cross-sectional study and due to the lack of the control group within the study. Therefore, prospective studies are still needed, with a control group and larger number of subjects, in attempt to exclude possible confounding factors and strengthen the results.

In conclusion, this study suggests that LAP and VAI are sensitive markers for screening and prevention of metabolic syndrome and IR in PCOS. In addition, LAP may be more sensitive than VAI in this identification, just as the A/G ratio may be more sensitive than body fat percentage. Therefore, this study has identified that these low-cost mathematical methods can aid in the medical routine.
